# Dynamic changes of extracellular vesicles during zebrafish organogenesis

**DOI:** 10.1186/s12964-025-02053-x

**Published:** 2025-02-03

**Authors:** Linda-Marie Mulzer, Tim Felger, Luis E. Muñoz, Gesa Engl, Heiko Reutter, Mario Schiffer, Leila Pourtalebi Jahromi, Fanni Annamária Boros, Friederike Zunke, Philipp Arnold, Alina C. Hilger

**Affiliations:** 1 Department of Pediatrics and Adolescent Medicine, Division of Neonatology and Pediatric, Intensive Care University Hospital Erlangen, Erlangen, Germany; 2 Department of Internal Medicine 3 Rheumatology and Immunology, Friedrich-Alexander- University Erlangen-Nürnberg (FAU) and Universitätsklinikum Erlangen, Erlangen, 91054 Germany; 3 Deutsches Zentrum für Immuntherapie (DZI), Friedrich-Alexander-Universität Erlangen- Nürnberg (FAU), Universitätsklinikum Erlangen, Erlangen, 91054 Germany; 4 Department of Internal Medicine 4 Nephrology, Friedrich-Alexander-University Erlangen- Nürnberg (FAU) and Universitätsklinikum Erlangen, Erlangen, 91054 Germany; 5 Department of Biology, Friedrich-Alexander-University Erlangen (FAU), Erlangen, Germany; 6 Department of Molecular Neurology, University Hospital Erlangen, Friedrich-Alexander- University Erlangen-Nürnberg (FAU), Erlangen, 91054 Germany; 7 Institute of Anatomy, Functional and Clinical Anatomy, Friedrich-Alexander-University Erlangen-Nürnberg (FAU), Erlangen, 91054 Germany; 8 Department of Pediatrics and Adolescent Medicine, University Hospital Erlangen, Erlangen, Germany; 9 Research Center on Rare Kidney Diseases (RECORD), University Hospital Erlangen, Erlangen, Germany

## Abstract

Extracellular Vesicles (EVs) play a crucial role in cell differentiation. Despite its role as a well-established vertebrate model, little is known about EVs during zebrafish embryogenesis. This study investigates EVs during zebrafish embryogenesis, analysing size- and concentration-changes over time.

Wild-type AB strain zebrafish larvae (zfl) were collected at 24, 48, 72, and 96 h post fertilization (hpf) and homogenized. EVs were isolated and characterized using flow cytometry, negative staining transmission electron microscopy (TEM), nanoparticle tracking analysis (NTA), and Western Blot.

Flow cytometry and TEM showed a high purity of the samples. Small EVs (sEVs) and large EVs (lEVs) were differentiated using NTA, showing that zfl use different types of EVs during embryogenesis. It was observed that the total EV number increased significantly over the first 72 hpf, but not proportionally to zfl growth in length. Additionally, sEV size also increased significantly, with a maximum diameter at 72 hpf. Since most organs are formed during the first 72 hpf and from then on mainly maturation and growth occur, the elevated number and larger size before 72 hpf suggests an important role of EVs during zebrafish organogenesis. Since EVs serve as cargo delivery platforms, the increase in size of sEVs may reflect the need for a higher transport capacity.

**Supplementary Information:**

The online version contains supplementary material available at 10.1186/s12964-025-02053-x.

## Introduction

Extracellular Vesicles (EVs) are cell membrane-enclosed nanoparticles that can be released from almost all cell types across the three domains of life (archaea, bacteria, and eukary) and cannot replicate on their own [1, 2]. EVs belong to the broader group of Extracellular Particles (EPs), an umbrella term for all particles outside the cell, which can then be further characterized into Vesicular and Non-Vesicular Extracellular Particles (NVEP). Within the group of Vesicular Extracellular Particles, Artificial Cell-derived-Vesicles (ACDVs), produced under laboratory conditions, such as cell disruption and Synthethic Vesicles (SVs), synthesized de novo, should be distinguished from EVs [3]. EVs can be classified according to their size, biogenesis and subcellular origin [2]. However, there is a large overlap in size of the different forms of EVs, therefore the determination of size alone does not prove their origin and can only be used as an additional criterion. Apoptotic bodies with over 1.000 nm represent the largest group of EVs and are released during cell apoptosis (controlled cell death) [4]. Microvesicles (also known as ectosomes or microparticles) have an approximate size range from 100 nm up to 1.000 nm and are released by living cells through outward blebbing of the cell membrane [5] they can be counted to the group of large EVs (lEVs). The term Exosome refers to EVs that originate from internal compartments of the cell and are released through multivesicular bodies (MVBs) that fuse with the cell membrane thus, belonging to the endosomal/autophagy system. Exosomes are typically smaller, ranging from 30 to 100 nm and can be counted as small EVs (sEVs) [2, 6, 7].

EVs are released in both physiological and pathophysiological conditions and play a major role in intercellular communication and extracellular organ cross-talk [8–10]. EVs are involved in cell migration and differentiation, an essential process for the development, embryogenesis, and maintenance of multicellular organisms [11]. Zebrafish (*Danio rerio*) are broadly-used vertebrate model organisms [12] with extensive applications in translational research on human diseases. They are specifically suitable models for investigating developmental defects because the entire embryonic development happens *ex utero* within a short period of time, providing convenient access to different developmental states. Moreover, the high transparency of the developing embryo and larvae allows direct microscopic in vivo visualization of embryonic structures [13]. However, the current literature and online databases (e.g. ExoCarta and Vesiclepedia) are limited about the role of EVs in zebrafish embryogenesis and organ maintenance.

Recently, a review of the literature by Zhao et al. summerized the available information on EVs extracted from different aquatic species. This review overview of EVs from different tissues, details of extraction methods, various identification and characterization approaches, contents analysis of EVs, and their application [14]. It shows, that most of the reported studies focus on sEVs (< 100 nm) and use 0.1 μm filters as part of the isolation protocol, which excludes lEVs, despite the fact that the size range of EVs could be up to 1.000 nm [15, 16]. Recently, specific EV-marker-labelled models of zebrafish have been used to investigate the biogenesis and distribution of EVs in vivo [17–19]. Furthermore, EVs have been reported from fin blastema [20], zebrafish osteoblasts [21], and from in vitro zebrafish cell lines [22]. However, studies on whole zebrafish embryos comprising the first 96 hpf of their embryonic development in a timeline-dependent manner are missing.

Therefore, our present study investigated EVs during whole zebrafish embryogenesis and larval development. We isolated lEVs and sEVs from I) whole zebrafish larvae (zfl) and II) the cultured zfl medium during four time points of embryogenesis. This was followed by EV characterization regarding their size and concentration, the essential parameters to be reported in EV research according to the Minimal Information for Studies of Extracellular Vesicles guidelines (MISEV 2023) [3]. To characterize and differentiate lEVs and sEVs we used flow cytometry, negative-stain transmission electron microscopy (TEM), nanoparticle tracking analysis (NTA), and Western Blotting (WB). We described lEVs based on their size as well as the presence of phosphatidylserine in the outer leaflet of the vesicles’ membrane, which is also in agreement with previous studies [23]. Using WB we showed that sEVs and lEVs express Alix. The change in EV populations we observed in the 96 hpf time window suggests that EVs play an important role in zebrafish organogenesis.

## Materials and methods

### Zebrafish husbandry

Zebrafish were kept according to the national regulations and recommendations by Westerfield [24] in our fish facility in Erlangen, Germany. Zfl of wild-type AB strains were obtained by natural spawning at 28 °C and raised in standard E3 solution at 28 °C. All national and institutional guidelines for experimenting on laboratory animals were strictly followed.

### Isolation and EV storage

**Fig. 1:**
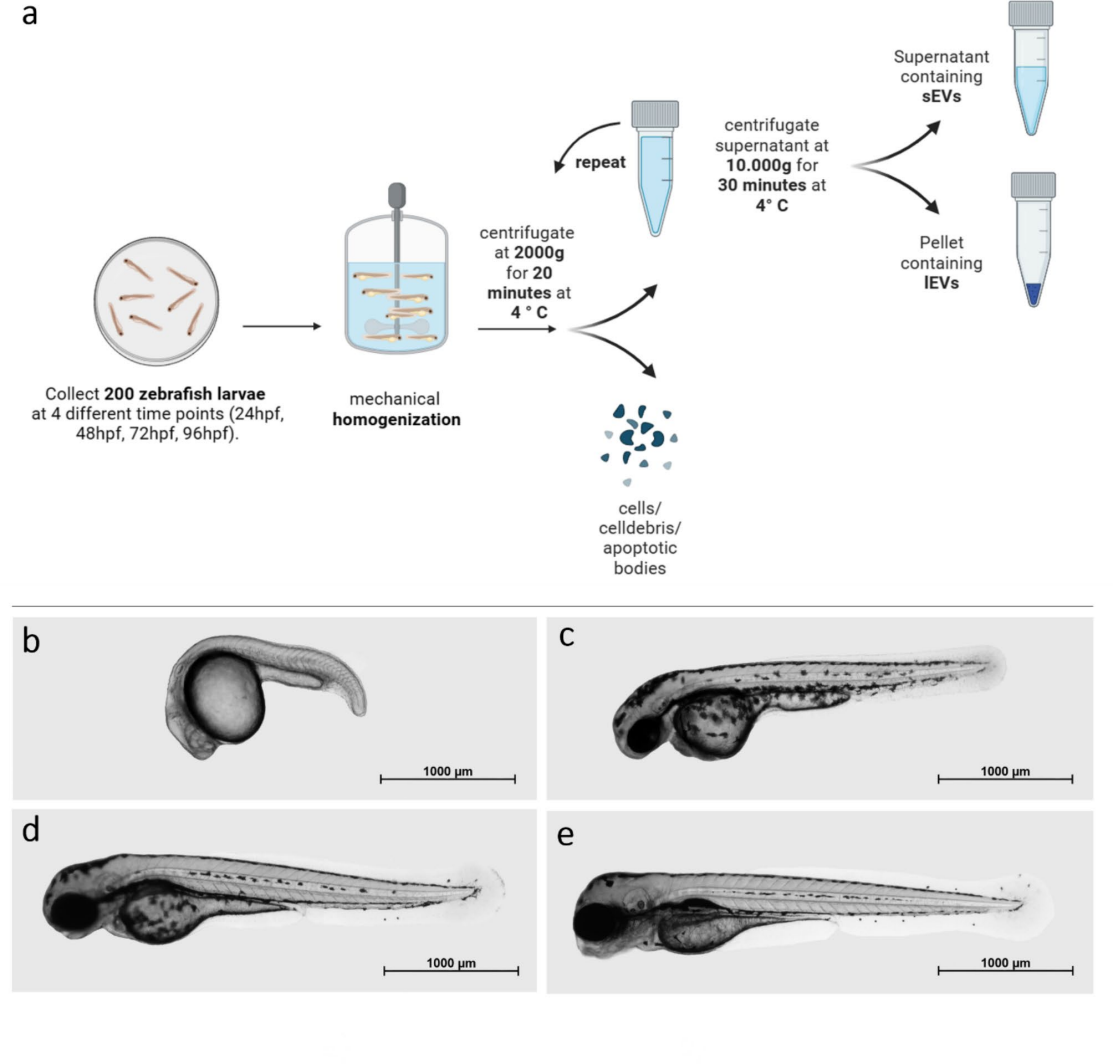
Protocol of EV isolation and zebrafish larvae development: **a:** Isolation protocol for whole zfl sEVs and lEVs by differential centrifugation. **b-e**: Zfl development; Imaging was performed at the 4 observation time points in 2% low-melting agarose (peqLab, Erlangen, Germany) using an Axio Observer microscope system (Zeiss, Jena, Germany), magnification 2,5x. **b:** 24 hpf, **c:** 48 hpf, **d:** 72 hpf, **e:** 96 hpf

Wild-type zebrafish embryos were cultured in E3 Medium at 28° C and collected at 24, 48, 72, and 96 hpf. Thereafter, 200 zfl per sampling time point were washed with E3 Medium and homogenized using a manual mechanical homogenizer (Squisher-Single, Zymo Research, Freiburg, Germany). For each time point, four independent samples were collected (200 zfl per sample). EV enrichment protocol was performed in reference to Thery et al. [25] by applying differential centrifugation. Cells, cell debris, and apoptotic bodies were depleted from the homogenized zebrafish lysate in two centrifugation steps, each at 2.000 ×g for 20 min at 4° C. The supernatant was then diluted with Ringer’s Solution (B. Braun, Melsungen, Germany) in a ratio of 1:100 and centrifuged at 10.000 ×g for another 30 min at 4° C. Next, this homogenized zfl supernatant (contains sEVs) and the homogenized zfl pellet (contains lEVs) were stored at -80° C in polypropylene tubes in a vertical position until further use as recommended [26]. The isolation protocol is demonstrated in Fig. 1a. The additional, in the original published article not used Ringer`s Solution was examined as a negative control in the TEM, NTA and flow cytometry analyses. No particles/ artefacts were found here. We were therefore able to use it without hesitation for dilution in the further isolation steps. Wild-type zfl medium was collected at 24 hpf, 48 hpf, 72 hpf, and 96 hpf and went through two centrifugation steps, each 2.000 ×g for 20 min at 4 °C to deplete cells, cell debris, and apoptotic bodies. The supernatant was vortexed and stored in a vertical position at -80 °C in polypropylene tubes until further use [26]. During all steps, an anti-static wrist strap was used as it is known that EVs have electrostatic properties [27].

### Flow cytometry

The homogenized zfl pellets from 24 hpf, 48 hpf, 72 hpf, and 96 hpf (*n* = 4 for each observation point) were analysed using flow cytometry. The homogenized zfl pellets were thawed in a water bath at 36° C for three minutes. After careful resuspension of the pellet in 1.000 µl Ringer’s Solution (B. Braun, Melsungen, Germany), AxV-FITC (FITC - conjugated recombinant Annexin V, Immunotools, Friesoythe, Germany) and FM4-64 (TermoFischer scientific, Waltham, MA, USA) were added immediately and incubated at room temperature for 30 min. Antibody concentration was determined in trials with 72 hpf homogenized zfl pellets. As recommended, unlabeled samples served to determine the background fluorescence [28]. Swarming effect was excluded through a dilution trial [29]. Dyes were centrifuged at 16.000 ×g for 15 min immediately before use to avoid agglutination and therefore, false positive events during flow cytometry.

The same protocol was also used for staining and flow cytometric analysis of EVs in wild-type zebrafish larvae medium collected at 72 hpf.

**Fig. 2:**
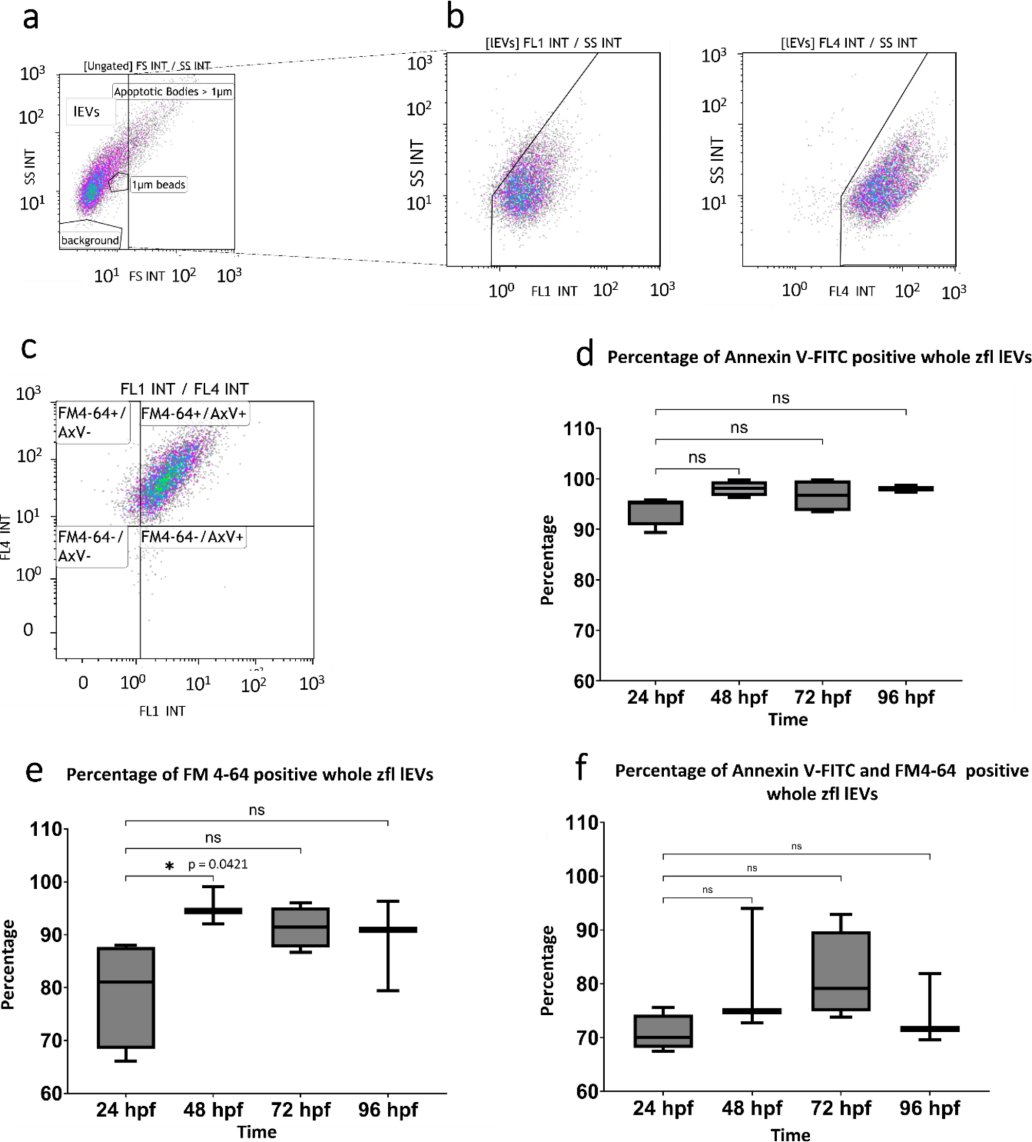
Characterization of whole zfl lEVs using flow cytometry: Gating strategy flow cytometry. **a**: First we determined the lEV gate by size using a forward scatter below the size of 1 micrometer polystyrene beads (Sigma-Aldrich, Steinheim, Germany) and excluding background signals as shown in the side scatter versus forward scatter plot. **b**: Further, a polygonal gate was used to exclude events with a higher side scatter, which might be aggregated lEVs. This can be seen in the scatter plots selected for the respective fluorescence: side scatter versus FL1 and side scatter versus FL4. AxV-FITC and/or FM4-64 were added to resuspended 10.000 g homogenized whole zfl pellets, incubated and measured directly and undiluted with flow cytometry. The expression of AxV-FITC and FM4-64 was analysed within these defined lEV gates. Gates defining the respective dye positive fraction were drawn in respect to an obviously negative fraction within the same measurement **c**: Double positive lEVs were gated in the same way and then analysed in 4 quadrants according to their positivity. FM4-64+/AxV-: lEVs negative for AxV-FITC but positive for FM4-64. FM4-64+/AxV+: lEVs showing positivity for both dyes FM4-64 and AxV-FITC. FM4-64-/AxV-: lEVs neither positive for AxV-FITC nor FM4-64. FM4-64-/AxV+: lEVs negative for FM4-64, but positive for AxV-FITC. **d**: Percentage of AxV + whole zfl lEVs at the 4 observation time points (24 hpf, 48 hpf, 72 hpf, 96 hpf), *n* = 4. **e**: Percentage of FM4-64 + whole zfl lEVs at the 4 observation time points (24 hpf, 48 hpf, 72 hpf, 96 hpf), *n* = 4. **f**: Percentage of FM4-64 + and AxV + whole zfl lEVs at the 4 observation time points (24 hpf, 48 hpf, 72 hpf, 96 hpf), *n* = 4. All results are presented as box plots with median, minimum, maximum, and interquartile ranges. * *p* < 0.05

lEVs were gated by size using a forward scatter below the size of 1 micrometer polystyrene beads (Sigma-Aldrich, Steinheim, Germany) under the exclusion of background signals. Further, a polygonal gate was used to exclude events with a higher sideward scatter, which might be aggregated lEVs (Fig. 2a). Fluorescent positive events were defined by their differentiation from antigen-negative events in stained samples and gated accordingly (Fig. 2a-b). Samples incubated with AxV-FITC and EDTA as a calcium chelator served as an additional negative control for the calcium-dependent AxV-FITC. Additionally, samples containing only Ringer’s Solution (B. Braun, Melsungen, Germany) and AxV-FITC and/or FM 4–64 served as negative controls. Samples were measured undiluted with a Gallios flow cytometer (Beckman Coulter, Brea, CA, USA). Excitation for fluorescence was at 488 nm and emitted fluorescence was recorded on the FL1 sensor (525/38nm BP Filter) as the AUC of FL1 and on the FL4 sensor (695/30nm BP Filter) as the AUC of FL4. Data analysis was performed with Kaluza software version 1.5 (Beckman Coulter, Brea, CA, USA).

### Nanoparticle tracking analysis (NTA)

The EVs in homogenized zfl pellets and supernatant collected at 24 hpf, 48 hpf, 72 hpf, and 96 hpf (*n* = 4 for each time point) underwent analysis using a nanoparticle tracking analysis (NTA) method. In this method, the nanoparticles scatter a laser beam, and a high-sensitivity camera tracks the particles’ Brownian motion by recording this scattered light. Then, by analysing the speed and the pattern of this motion, the hydrodynamic diameter of the particles is calculated using the Stokes-Einstein equation. The concentration is also calculated based on the number of detected particles in the NTA’s cell with known volume, the corresponding unit is particles/millilitre. Before the NTA measurements, the samples, previously stored at − 80 °C, were thawed and diluted in Ringer’s Solution (B. Braun, Melsungen, Germany). Particle yield and size distribution profiles were determined using a ZetaView^®^ PMX-220 (Particle Metrix, Germany), following the manufacturer’s recommended settings for EV analysis. Briefly, measurements were conducted with a 488 nm laser, in scatter mode, at 25 °C, employing a camera sensitivity of 80%, a shutter duration of 100 ms, and a frame rate of 30. The results were analysed using the ZetaVIEW software.

### Transmission electron microscopy (TEM)

TEM was performed on lEV and sEV samples previously stored at -80 °C. Negative staining TEM was performed according to Arnold et al. [30]. In brief, three microliters of sample were added to freshly negative glow discharged carbon-coated EM grids (Electron Microscopy Sciences, Hatfield, United States). The excess liquid was removed immediately with filter paper. Afterwards, the EM grid was washed with 2% aqueous uranyl acetate solution twice (Merck Millipore, Billerica, MA, United States), blotted with a filter paper once more, and left to air dry. The micrographs were collected on a JEOL 1400 Plus TEM (JEOL Germany, Munich, Germany) operating at 120 kV with a nominal magnification of 30.000x.

### Western Blotting (WB)

For SDS-PAGE and WB analysis, sEV and lEV samples of the same hpf were loaded onto pre-cast Bis-Tris 4–12% gradient gel (Invitrogen, Bolt Mini-Protein-Gel, cat. no.: NW04125BOX). The loaded protein amount ranged from 2 to 4.5 µg, and was equal in case of sEVs and lEVs. As control 4.5 µg zebrafish protein isolated from 72 hpf larvae (see supplementary material) was loaded, as molecular weight marker pre-stained protein marker was used (PageRuler Plus Prestained protein ladder, cat. no.: 26619). The gel was set up in 1x MES buffer and run at constant 120 V. The proteins separated based on their molecular size were then transferred onto a PVDF membrane (Immobilion-FL, 0.45 μm pore size, Millipore, cat. no.: IPFL00010) with constant 30 V for 1 h 10 min. Following transfer, the membrane was fixed in 0.4% PFA by constant rotation for 20 min at room temperature. The fixation was followed by 3-times 5 min washing steps with MilliQ water, after which the membrane was stained with Ponceau S (Sigma, cat. no.: P7170-1 L). The membrane was de-stained with 1x transfer buffer containing 20% MeOH, and blocked in blocking buffer (Licor, cat. no. 927-60001) for 1 h at room temperature. For antibody incubation the primary antibody (rabbit Alix monoclonal antibody, clone E6P9B, Cell Signaling Technology, cat. no.: 92880 S, lot:1) was diluted 1:500 in antibody diluent (Licor, cat. no.: 927-65001) and incubated overnight at 4 °C. Following incubation, the membrane was washed with 1xTBS containing 0.1% Tween20 (TBS-T) (3-times 10 min each), and incubated with fluorescently labelled secondary antibody (IRDye 800CW donkey anti-rabbit, Licor cat. no.: 926-32213) diluted 1:10.000 in TBS-T containing 2% fishgelatine for 1 h at room temperature. After discarding the secondary antibody, the membrane was washed 3-times with TBS-T, and scanned with Odyssey M scanner.

### ZFL microscopy

Zebrafish embryos were raised at 28 °C in a petri dish containing E3 Medium until they reached the indicated developmental stages at 24 hpf, 48 hpf, 72 hpf, or 96 hpf. Immobilisation was reached by E3 Medium containing168 mg/L Tricaine and imaging was performed in 2% low-melting agarose (peqLab, Erlangen, Germany) using an Axio Observer microscope system (Zeiss, Jena, Germany), magnification 2,5x (Fig. 1b-e).

### Statistical analysis

Statistical analysis was carried out with Prism 6.0 (GraphPad Software Inc., La Jolla, CA, USA). All data passed the Shapiro-Wilk-Lognormality test (alpha = 0.05). Then, different groups were compared through ANOVA followed by Dunnett`s multiple comparison test. No outliers were defined using the ROUT method (Q = 1%). Results are presented with boxplots showing medians, minimum, maximum, and interquartile ranges. Results were defined significant with a *p*-value below 0.05.

## Results

EVs during zfl development were characterized by changes in size, number, shape and surface marker within the first 96 hpf.

### Flow cytometry

The flow cytometric analyses revealed that 89.4 -99.8% of the events gated as lEVs were positive for AxV-FITC. The AxV-FITC staining was fully reversible with EDTA. The fraction of AxV-FITC positive lEVs increased with progressing development of larvae but without reaching the level of significance. 66.1- 99.1% of the flow cytometry events gated as lEVs were FM4-64 positive. The percentage of FM4-64 positive lEVs increased over time, with statistical significance between 24 hpf and 48 hpf (*p* = 0.0421). It is noticeable that 67.5-94% of the events gated as lEVs were stained double positive for Annexin V-FITC and FM4-64 (Fig. 2c-e), whose fraction showed a tendency to increase within the first 72 hpf without reaching the level of significance. The mean fluorescence intensity (MFI) of FM4-64 positive lEVs also showed no changes over time, while the MFI of AxV-FITC positive lEVs abated. This effect was significant at 72 hpf and 96 hpf compared to 24 hpf (*p* = 0.0381, *p* = 0.0054) (supplementary Fig. 1).

### NTA

**Fig. 3:**
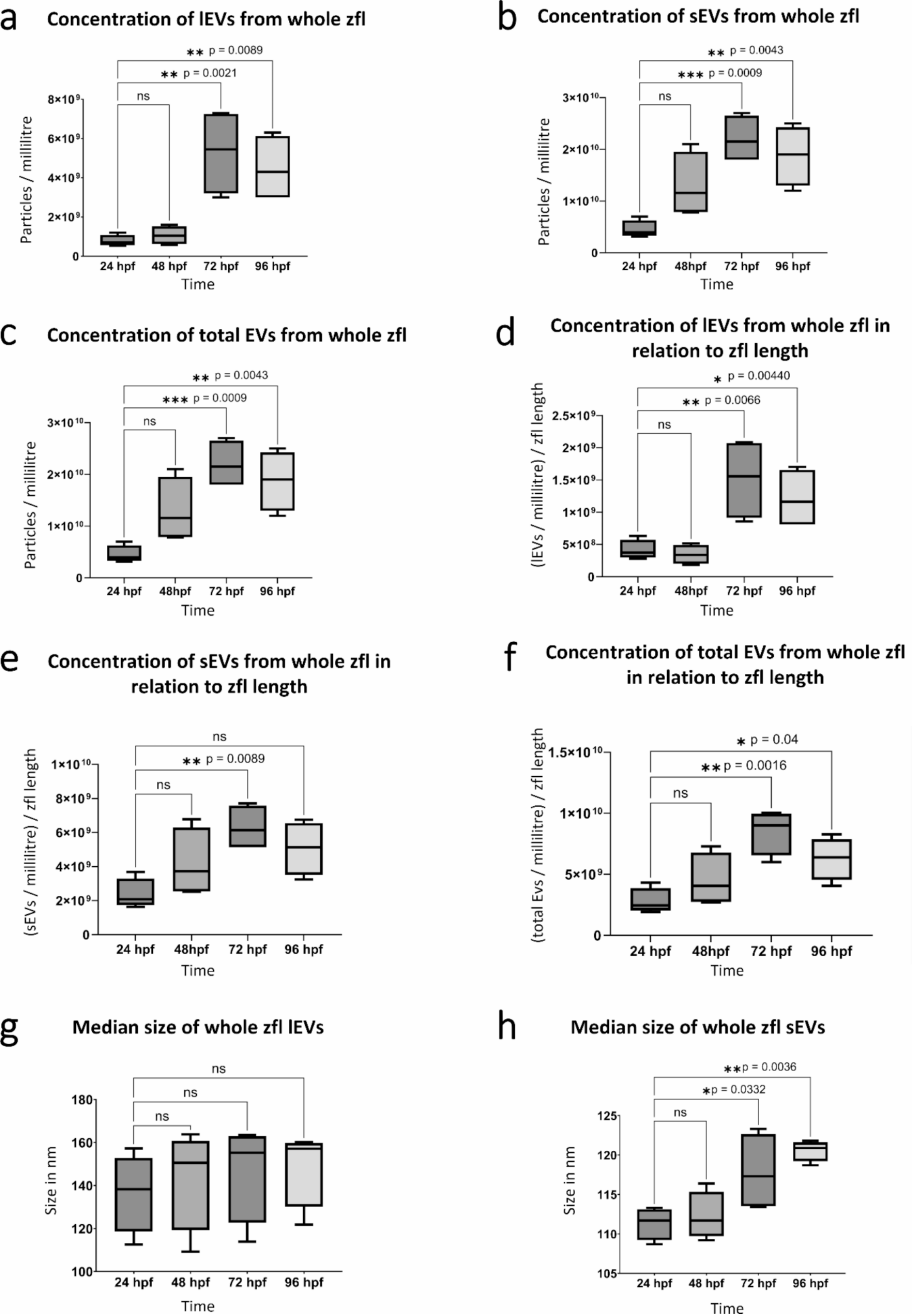
Determination of size and concentration of whole zfl EVs: **a-f**: Quantity of zfl sEVs, lEVs, and total EVs during the first 96 hpf. Concentration of EVs (particles/millilitre) was determined using NTA. **a**: Particles/millilitre of zfl lEV samples at the 4 observation time points (24 hpf, 48 hpf, 72 hpf, 96 hpf), *n* = 4. **b**: Particles/millilitre of zfl sEV samples at the 4 observation time points ( 24 hpf, 48 hpf, 72 hpf, 96 hpf), *n* = 4. **c**: Particles/millilitre of zfl lEV samples and sEV samples (= total EVs) at the 4 observation time points (24 hpf, 48 hpf, 72 hpf, 96 hpf), *n* = 4. **d**: particles/millilitre of zfl lEV samples at the 4 observation time points (24 hpf, 48 hpf, 72 hpf, 96 hpf), *n* = 4, divided through the length of the zfl at the respective time point. **e**: Particles/millilitre of zfl sEV samples at the 4 observation time points (24 hpf, 48 hpf, 72 hpf, 96 hpf), *n* = 4, divided through the length of the zfl at the respective time point. **f**: Particles/millilitre of zfl lEV and sEV samples (= total EVs) at the 4 observation time points (24 hpf, 48 hpf, 72 hpf, 96 hpf), *n* = 4, divided through the length of the zfl at the respective time point. All results are presented as box plots with median, minimum, maximum, and interquartile ranges. * *p* < 0.05. **g-h**: Median size of zfl sEVs and whole zfl lEVs during the first 96hpf. Size was determined using NTA. **g**: Median size of whole zfl lEVs at the 4 observation time points (24 hpf, 48 hpf, 72 hpf, 96 hpf), *n* = 4. **h**: Median size of whole zfl sEVs at the 4 observation time points (24 hpf, 48 hpf, 72 hpf, 96 hpf), *n* = 4. All results are presented as box plots with median, minimum, maximum, and interquartile ranges. * *p* < 0.05

The median sizes of the two EV fractions (lEVs and sEVs), isolated via differential centrifugation, differed at each of the investigated time points (supplementary Fig. 2). The NTA results showed a gradual increased concentration of sEVs, lEVs, and total EVs (Fig. 3a-c), not significant within the first 48 h, but clearly significant at 72 hpf and 96 hpf compared to 24 hpf (sEVs: *p* = 0.0009, *p* = 0.0043; lEVs: *p* = 0.0021, *p* = 0.0089; all EVs: *p* = 0.0002, *p* = 0.0024). It is worth noting that the concentration of sEVs, lEVs, and total EVs showed a slight reduction (statistically insignificant) from 72 hpf to 96 hpf. We could also see a similar trend by correlating the number of sEVs, lEVs, and total EVs per millilitre and the length of the zfl at the respective developmental time points (Fig. 3d-f). Standard size of zfl at the respective time points were adopted from zfin.org [31] and matched the size of zfl used in our study (supplementary Table 1). The number of sEVs, lEVs, and total EVs per millilitre in relation to the respective size of the zfl in mm at the observed hpf increase over time. The increase was not significant within the first 48 hpf, but at 72 hpf and 96 hpf numbers of sEVs, lEVs, and total EVs increased significantly in relation to the length of zfl compared to 24 hpf. The number of sEVs, lEVs, and total EVs per millilitre in relation to the size of the zfl in mm at the respective developmental time point showed a slight decrease from 72 hpf to 96 hpf (Fig. 3d-f). The median size of lEVs remained the same over time, ranging from 130 nm to 164 nm. However, sEVs became significantly larger over time, with the median size ranging from 109 to 123 nm. This increase in size was not significant within the first 48 hpf, but at the time of 72 hpf and 96 hpf sEVs increased significantly (*p* = 0.0332, *p* = 0.0036) in size compared to EV size at 24 hpf (Fig. 3g-h).

### TEM

**Fig. 4:**
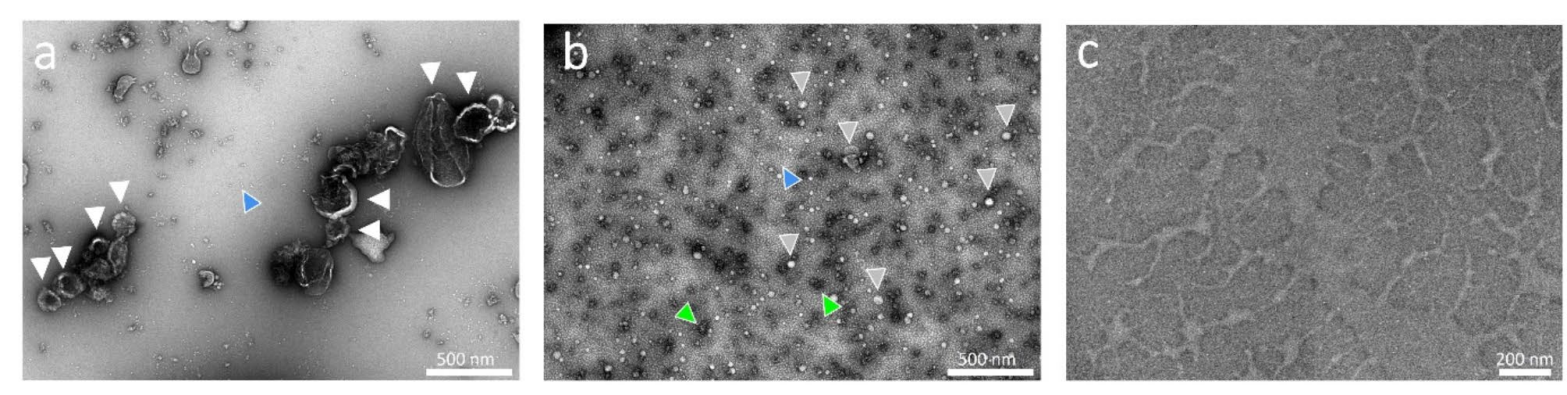
Morphology characterization of whole zfl sEVs and lEVs by TEM: The micrographs were collected on a JEOL 1400 Plus TEM (JEOL Germany, Munich, Germany) operating at 120 kV with a nominal magnification of 30.000x. **a**: 10.000 g homogenized whole zfl pellet at 30k magnification: cup shaped lEVs (white arrowhead), in the background some triangular shaped protein (blue arrowhead). The white bar in the lower right quadrant of the image corresponds to 500 nm. **b**: 10.000 g homogenized whole zfl supernatant at 30k magnification: sEVs of around ~ 70–100 nm (some depicted with grey arrowheads), the background is covered with triangular shaped protein (blue arrowhead) and some proteins aggregates appear (two marked with green arrowheads). The white bar in the lower right quadrant of the image corresponds to 500 nm. **c**: Zfl medium at 72 hpf at 30k magnification: no EVs, background with staining artefact. The white bar in the lower right quadrant of the image corresponds to 200 nm

TEM analysis of the homogenized zfl pellet showed cup-shaped lEVs with a size around 150–250 nm (Fig. 8a, white arrowhead). In the background, some triangular-shaped protein can be seen. This is presumably a heat shock protein from larval serum (Fig. 4a, blue arrowhead). In the homogenized zfl supernatantsEVs were enriched, ranging in size from 70 to 100 nm (Fig. 4b, some with grey arrowheads). Additionally, we could not only identify protein aggregates (green arrowheads) but also realized that the background is covered with the triangular proteins we had seen in the pellet samples, but in a larger quantity. Finally, in the ZF-Medium, neither proteins or cell debris nor EVs were detected (Fig. 4c).

### Western Blotting

For both, sEVs and lEVs, the commonly used EV marker Alix showed strong signals using immunoblot analyses (supplementary Fig. 3). Alix was shown to be positive in zf EVs before and is an established marker for EVs involved in their biogenesis via the the syntenin-Alix-ESCRT-III pathway [32]. The signal intensity of the lEVs was more intense than the sEVs. The negative control zfl medium showed no signal in the expected area. Also, whole zebrafish protein showed a different band pattern, indicating antibody specificity towards EVs.

## Conclusion/Discussion

To the best of our knowledge, the present study is the first to characterize and differentiate EVs from the whole zfl within the first 96 hpf. After EV isolation by differential centrifugation, flow cytometry was performed for the lEV population to investigate the sample’s purity. We could show, that 89–99.8% of the events detected in the lEV gate contained phosphatidylserine, 66 − 99% of the events showed a lipid bilayer, and 68 − 95% of the events expressed phosphatidylserine and were built of a lipid bilayer confirming the purity of the lEV samples, based on the double positivity. The maximum number of lEVs was detected at 72 hpf. Since the presence of AxV negative lEVs has also been reported [33], the total amount of lEVs might be underestimated. Negative events could be liposomes, cell debris, parts of apoptotic bodies, and aggregated dyes, therefore centrifugation of the dyes right before usage was assessed to avoid the latter. To provide further evidence of the EV nature of the isolated particles, we conducted a WB analysis and found strong signals of Alix, a positive EV-marker [19] for both sEVs and lEVs. Nevertheless, through the process of mechanical homogenization we cannot completely exclude the presence of ACDV fractions. However, it shoul be assumed that the fraction of ACDVs does not depend on the developmental stages of the zfl but should rather remain unchanged for each time point analysed.

Next we used these pure EV samples for further NTA analysis to detect changes in size and concentration (particles/ml) over time. We applied NTA at 24 hpf, 48 hpf, 72 hpf, and 96 hpf to demonstrate differences in size between lEV and sEV fractions (24 hpf *p* = 0.03411; 48 hpf *p* = 0.03992; 72 hpf *p* = 0.04756; 96 hpf *p* = 0.02092). Our findings indicate that at least two distinct populations of EVs exist in zfl that might fulfill specific functions during development especially organogenesis. Additionally, NTA showed a significant increase of the total EV concentration, comprising sEVs and lEVs, over the first 72 hpf. Although the concentration of sEVs and lEVs increases from 24 to 72 hpf of the developing zfl, this increase is not proportional to the increase in length of the zfl during this developmental period, which means that we observed more increase of EVs as it would be expected just by the increase in length of the growing zfl. According to our results, the total number of EVs increased by factor 5.6, whereas the length of the zfl grows only by 1.9 during this period (supplementary Fig. 4). This non-linear relationship between growth and EV release might argue for a functional role of the distinct population of EVs as well as total EVs during the developmental processes. At 72 hpf we observed the highest number of total EVs in the developing zfl. Interestingly, the total number of EVs decreased slightly after 72 hpf. Considering that most organ systems have been formed in the developing zfl during the first 72 hpf [24] and mainly maturation and growth occur thereafter, we assume that the elevated EV number prior to 72 hpf fulfills an important role during organogenesis and development [34, 35].

Our data demonstrated that not only the concentration of all EV populations increase during embryonic zfl development, but also the size of sEVs increase significantly reaching a maximum at 72 hpf. The increase in size might be due to a higher metabolic activity of cells during the developmental stage of 24–72 hpf. Since EVs play a crucial role as intercellular cargo carriers (e.g. proteins, lipids, or nucleic acid components), larger vesicles may reflect a higher transport capacity [8]. A similar trend has been reported for bone marrow-derived macrophages (BMDMs) of mice, whose EVs become larger after being stimulated [36]. Nevertheless, scarce information is available about the size of EVs during embryogenesis, specifically when it comes to the correlation between the size and particular functions.

The NTA results were confirmed with TEM studies according to the guidelines of MISEV 2018. While TEM images of organ-specific or zf cell line-specific EVs had been reported before [22], to the best of our knowledge, this is the first study to present TEM images of whole zfl EVs. In our TEM images we can clearly differentiate the distinct EV populations (lEV and sEV), thereby confirming the efficiency of the used isolation method in enrichment of the distinct EV populations. Furhter we could not see any lipid-co isolates in TEM images matching the homogeneous particle distribution in terms of size in the NTA analyses. Nevertheless apart from EVs, an abundant amount of a triangular protein was detected in both fractions (lEVs and sEVs), which were also reported in other studies with negative stain TEM technique [37]. Possibly, this protein could represent a Heat Shock Protein (HSP) [38] known to be important as an active partner in cell differentiation and organism morphogenesis [39]. Since no further downstream analyses (e.g. proteomic determination of EV contend) were conducted, these co-isolated proteins should not affect the statements made.

In summary, this is the first study to characterize the morphology and size range of whole zfl sEVs and lEVs using flow cytometry, Western Blot, NTA, and TEM. Our observations suggest that with the progressing organogenesis of the developing zfl, an increase in EV number and EV size is necessary to orchestrate the maturing zebrafish embryo. Nevertheless, extensive studies are required to assess and describe the subtypes of vesicles to unravel their physiological or pathophysiological functions.

## Electronic supplementary material

Below is the link to the electronic supplementary material.

Supplementary material 1

## Data Availability

No datasets were generated or analysed during the current study.
