# Supplementary material for: Dynamic changes of extracellular vesicles during zebrafish organogenesis
Source: Cell Commun Signal. 2025 Feb 3;23:60. doi: 10.1186/s12964-025-02053-x (PMC11789338; doi:10.1186/s12964-025-02053-x)
Supplement: Supplementary file 1 — Supplementary material 1 [file 12964_2025_2053_MOESM1_ESM.docx]

## **Supplement**

## **Supplementary Figure 1**


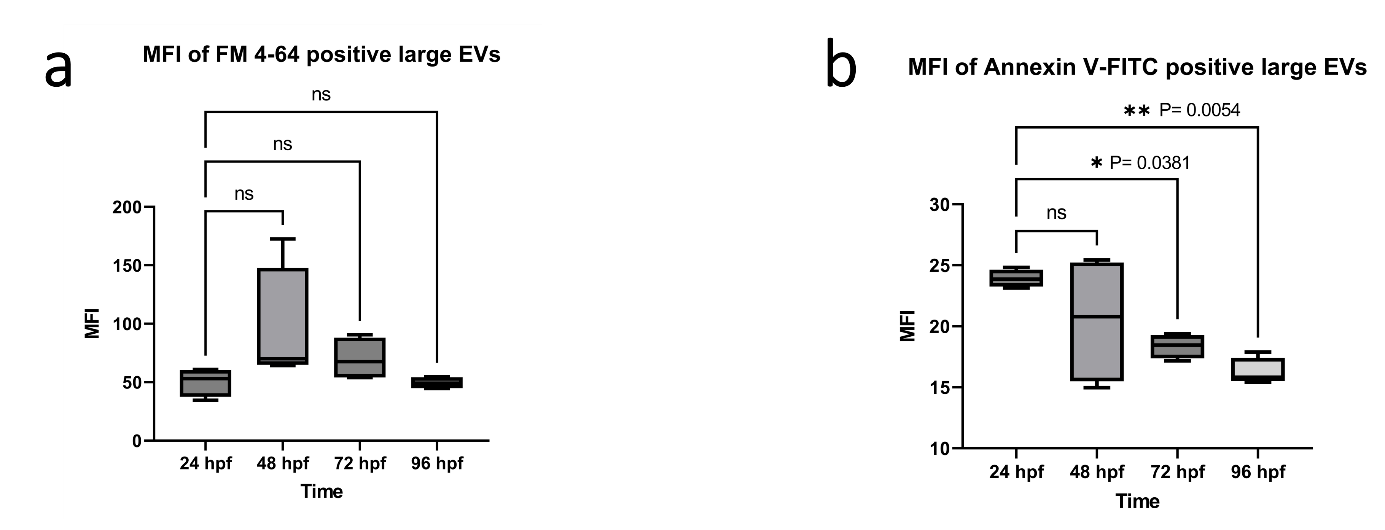


## **Supplementary Figure 1** AxV-FITC and/or FM4-64 were added to resuspended 10.000 g homogenized whole zfl pellets, incubated and measured directly and undiluted with flow cytometry. lEVs were defined by a gate that excluded background events and events with a higher forward scatter than 1µm polystyrene beads. The expression of AxV-FITC and FM4-64 was analyzed within this defined lEV gate. Gates defining the respective dye positive fraction were drawn in respect to an obviously negative fraction within the same measurement. Further, a polygonal gate was used to exclude events with a higher side scatter, which might be aggregated lEVs. **a:** MFI (mean fluorescence intensity) of AxV+ whole zfl lEVs at the 4 observation time points (24 hpf, 48 hpf, 72 hpf, 96 hpf), n=4. **b:**MFI of FM4-64+ whole zfl lEVs at the 4 observation time points (24 hpf, 48 hpf, 72 hpf, 96 hpf), n=4.

## **Supplementary Figure 2**


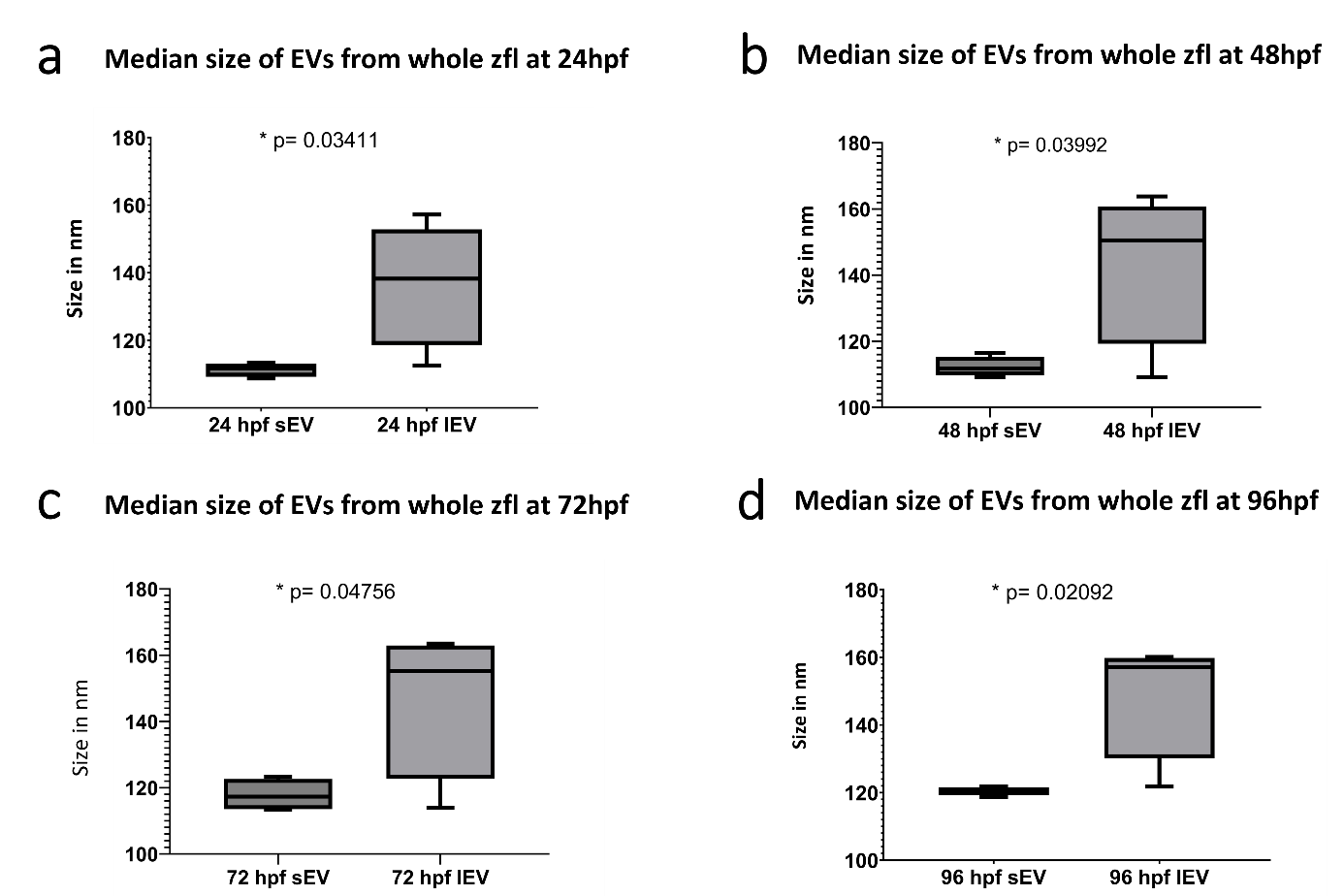


**Supplementary Figure 2** Comparison of median sizes of sEV and lEV fractions from whole zfl using NTA at different time points. a: 24 hpf, n = 4. b: 48 hpf, n = 4. c: 72 hpf, n = 4. d: 96 hpf, n = 4. All results are presented as box plots with median, minimum, maximum, and interquartile ranges. * p < 0.05.

## **Supplementary Figure 3**


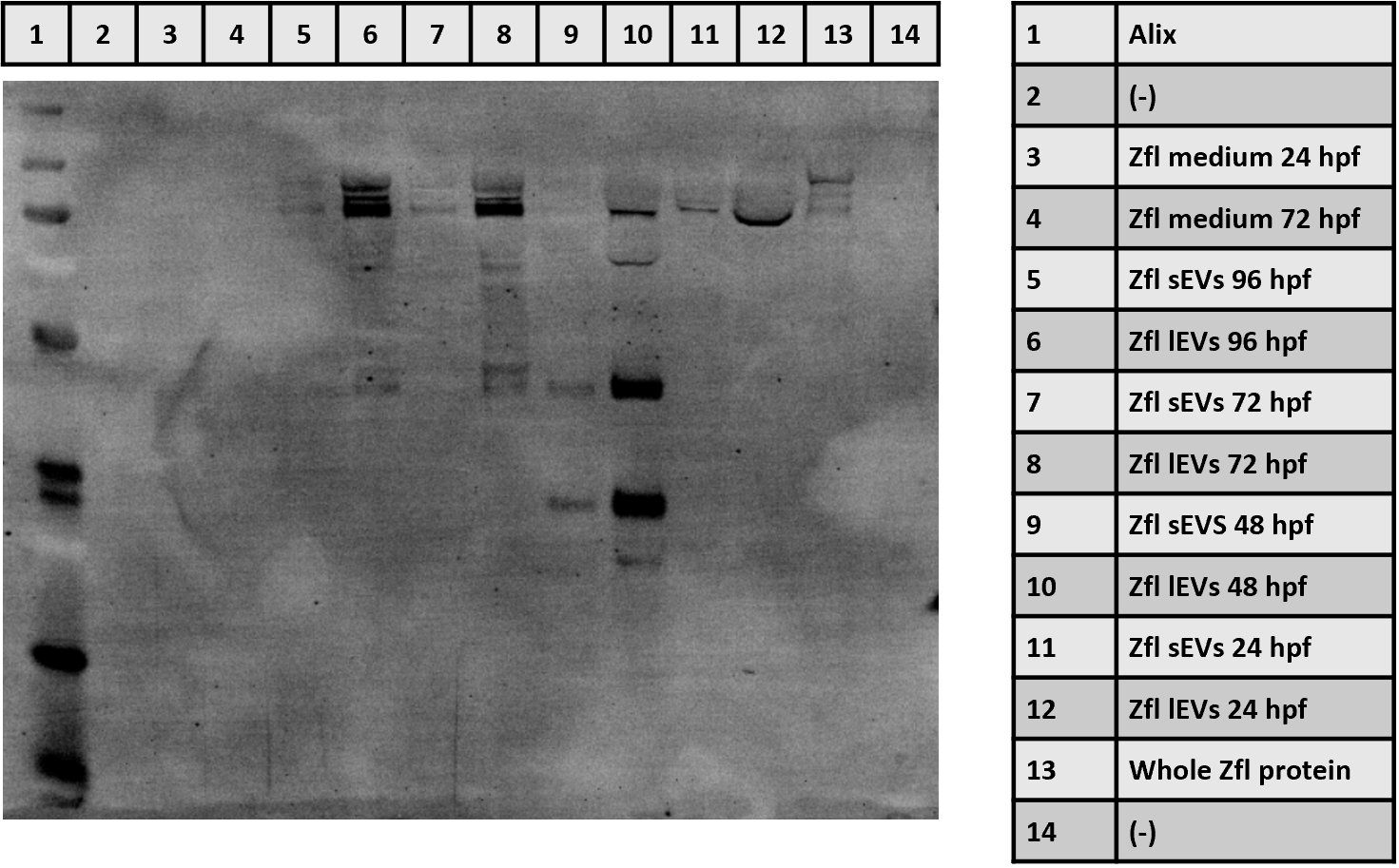


**Supplementary Figure 3** Western Blot analysis of whole zfl sEV and lEV samples from 24 hpf, 48 hpf, 72 hpf and 96 hpf. The signal intensity of the lEV samples (6, 8, 10, 12) was more intense than of the sEV samples (5, 7, 9, 11). The negative control zebrafish larvae medium (3, 4) showed no signal in the expected area. Also, whole zebrafish protein (13) showed a different band pattern, indicating antibody specificity towards EVs.

**Supplementary Figure 4**

**Supplementary Figure 4** Zfl length growth and increase of zfl total EVs over time are shown. Length and concentration at time point 24 hpf were used as baseline. (= 100%, 1.0).

## **Supplementary Table 1**

## **Comparison of zfl length reported from zfin.org with zfl used in this study**

|  | **Zfl used for this study** | **Values reported from zfin.org** |
| --- | --- | --- |
|  |  |  |
| **Age** | **Average size in mm** | **Average size in mm** |
| **24 hpf** | 1,933333333 | 1,9 |
| **48 hpf** | 3,066666667 | 3,1 |
| **72 hpf** | 3,5 | 3,5 |
| **96 hpf** | 3,75 | 3,7 |

## ***Supplementary Table 1*** Sizes of zfl reported from zfin.org in comparison to sizes of zfl used in this study. Mean sizes of zfl were meassured using zfl images performed at the 4 observation time points using an Axio Observer microscope system (Zeiss, Jena, Germany), magnification 2,5x as shown in supplementary figure 4. (n = 3 for each time point).

## **Extraction of whole zebrafish protein:**

Whole zebrafish protein from 72 hpf zfl was extracted using RIPA buffer (recipe below) which was supplemented with protease inhibitors before use. The zebrafish larvae were raised as described above till 72 hpf, then 40 zfl were collected, dechorionated, twice rinsed in Ringer`s Solution (B. Braun, Melsungen, Germany) and deyolked. Afterwards, the prepared larvae were transferred to a 1.5 ml Eppendorf tube, again rinsed 3 times in Ringer`s Solution (B. Braun, Melsungen, Germany) before removing all remaining fluid. To extract the protein, 400 µl RIPA buffer was added, followed by a 30 minute incubation on ice and homogenization thereafter. The homogenized sample was then incubated at 4°C in a shaker at full speed. Right after the incubation, the sample was centrifuged for 20 minutes at 4°C and 13.000 rpm. The liquid supernatant was equally divided in volume transferred into 1.5 ml Eppendorf tubes and frozen at -80°C until further use.

## **Recipe for RIPA buffer:**

1. Fill 80 ml Milli-Q® water in a fresh 100 ml bottle
2. Ad following materials:
   - 790 mg Tris (Tris(hydroxymethyl)aminomethan) (~ 50 mM)
   - 900 mg Natriumchlorid (~150 mM)
   - 1 ml Igepal CA-630 (1%)
   - 250 mg Sodium Deoxycholate (~ 6.5 mM or 0.25%)
   - 74.4 mg EDTA (~2.5 mM)
3. Adjust pH 7.4 with HCl/NaOH when everything is dissolved
4. Fill up to 100 ml with Milli-Q® water
5. Filter as sterile as possible
